# A novel locus (*CORD12*) for autosomal dominant cone-rod dystrophy on chromosome 2q24.2-2q33.1

**DOI:** 10.1186/1471-2350-12-54

**Published:** 2011-04-15

**Authors:** Gaël Manes, Maxime Hebrard, Béatrice Bocquet, Isabelle Meunier, Delphine Coustes-Chazalette, Audrey Sénéchal, Anne Bolland-Augé, Diana Zelenika, Christian P Hamel

**Affiliations:** 1 INSERM U1051, Institute for Neurosciences of Montpellier, (80 rue Augustin Fliche), Montpellier, (34091), France; 2 Université Montpellier 1, (2 rue Ecole de Médecine), Montpellier, (34060), France; 3 Université Montpellier 2, (Place Eugène Bataillon), Montpellier, (34095), France; 4 Genetics of Sensory Diseases, CHRU Gui de Chaulliac, (80 rue Augustin Fliche), Montpellier, (34295), France; 5 Centre National de Génotypage, (2 rue Gaston Crémieux), Evry, (91057), France

## Abstract

**Background:**

Rod-cone dystrophy, also known as retinitis pigmentosa (RP), and cone-rod dystrophy (CRD) are degenerative retinal dystrophies leading to blindness. To identify new genes responsible for these diseases, we have studied one large non consanguineous French family with autosomal dominant (ad) CRD.

**Methods:**

Family members underwent detailed ophthalmological examination. Linkage analysis using microsatellite markers and a whole-genome SNP analysis with the use of Affymetrix 250 K SNP chips were performed. Five candidate genes within the candidate region were screened for mutations by direct sequencing.

**Results:**

We first excluded the involvement of known adRP and adCRD genes in the family by genotyping and linkage analysis. Then, we undertook a whole-genome scan on 22 individuals in the family. The analysis revealed a 41.3-Mb locus on position 2q24.2-2q33.1. This locus was confirmed by linkage analysis with specific markers of this region. The maximum LOD score was 2.86 at θ = 0 for this locus. Five candidate genes, *CERKL*, *BBS5, KLHL23, NEUROD1*, and *SF3B1 *within this locus, were not mutated.

**Conclusion:**

A novel locus for adCRD, named *CORD12*, has been mapped to chromosome 2q24.2-2q33.1 in a non consanguineous French family.

## Background

Retinitis pigmentosa (RP, [MIM 268000]) is a genetically heterogeneous group of retinal photoreceptor degeneration characterized by night blindness and loss in the peripheral visual field, slowly progressing towards total blindness after several decades [1]. RP accounts for about 2/3 of the inherited retinal dystrophy cases [2]. In contrast to typical RP, also called rod-cone dystrophies (RCDs) because of primary involvement of rods, inverse RP or cone-rod dystrophies (CRDs) are pigmentary retinopathies characterized by first decrease in visual acuity and loss in the central visual field and lately by night blindness and loss in the peripheral visual field. CRDs are due to the primary degeneration of cone photoreceptors, followed by the secondary, or, sometimes, concomitant loss of rod photoreceptors [3]. Fourty nine genes and loci are responsible for non syndromic RP and 18 for non syndromic CRD (including 6 in common with RP and 4 with Leber congenital amaurosis) http://www.sph.uth.tmc.edu/Retnet. The three types of Mendelian inheritance are encountered in both RP and CRD.

Among the 18 CRD genes, ten (*GUCY2D, PITPNM3, GUCA1A, HRG4/UNC119, CRX, AIPL1, RIMS1, SEMA4A, PROM1 and PRPH2/RDS*) are found in autosomal dominant (ad) CRD, six (*ABCA4, RPGRIP1, RAX2, CORD8, ADAM9 *and *CERKL*) in autosomal recessive (ar) CRD and two (*RPGR *and *CACNA1F*) in X-linked CRD http://www.sph.uth.tmc.edu/Retnet. The prevalence of mutations for each gene in the CRD population is highly variable. *ABCA4*, which causes Stargardt macular dystrophy, is also a major gene for CRD, being responsible for 30-60% of arCRD cases [4-6]. In contrast, the overall prevalence of adCRD genes remains low, many of them being described in only one or a few cases. Only *CRX*, *GUCY2D *and *PRPH2/RDS *have been consistently reported in adCRD [7-10]. Yet, *CRX *was estimated to account for only 5-10% of adCRD cases and the prevalence of *GUCY2D *and *PRPH2/RDS *is unknown [11,12]. Therefore, there are probably other genes remaining to be discovered in adCRD.

In search for new genes responsible for pigmentary retinopathies, we recruited one large non-consanguineous French family with adCRD. This family was unlinked to any known adRP or adCRD locus and SNP genotyping revealed that it was linked to a new locus on chromosome 2, designated *CORD12*.

## Methods

### Clinical examination

**Figure 1:**
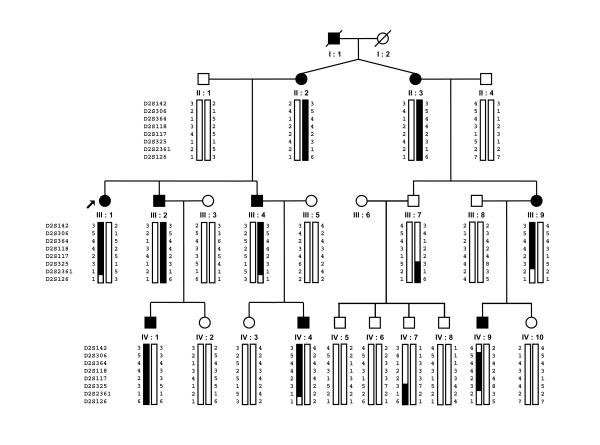
**Pedigree of family RP470 with autosomal dominant inherited cone-rod dystrophy (adCRD)**. Arrow indicates the index patient. Filled symbols represent members with adCRD and empty symbols represent unaffected patients. Haplotypes of microsatellite markers spanning the locus 2q24.2-2q33.1 are shown. Question marks indicate unknown alleles. Solid bars denote the haplotype that segregated with the disease phenotype.

Members of this large French non-consanguineous family (RP470) were identified with CRD which segregated as a dominant trait (adCRD). There were 9 affected patients out of 22 in 4 generations (Figure 1). Examination included assessment of visual acuity, slit lamp biomicroscopy, direct funduscopy and full field electroretinography. There was no evidence of extra-ocular signs of disease indicating that CRD was non syndromic.

### Genotyping of microsatellite markers and linkage analysis

Informed written consent and peripheral blood samples were obtained from 22 examined family members. The investigators followed the tenets of the Declaration of Helsinki. Genomic DNA was isolated from 10 ml peripheral blood leucocytes using standard salting out procedure [13]. The DNA samples were quantified by a spectrophotometer and diluted to 25 ng/μl for PCR amplification. PCR was carried out in a 25 μl final volume containing 50 ng genomic DNA, 5 picomoles of each primer, 0.2 mM dNTPs (MP Biochemicals), 2 mM MgCl_2_, PCR buffer and 1 unit of DNA polymerase (AmpliTaq Gold; Applied Biosystems, Foster city, CA). Initial denaturation at 95°C for 10 minutes was followed by 35 cycles of denaturation at 94°C for 30 seconds, specific annealing temperature for 30 seconds, and extension at 72°C for 1 minute. A final extension step was performed at 72°C for 10 minutes. The PCR products were diluted and mixed with Genescan 400HD ROX size standard and subsequently analysed on an Applied Biosystems 3130xL genetic analyser (Applied Biosystems, Foster city, CA).

Genotyping was performed using 2 to 3 polymorphic commercially available microsatellite markers from ABI PRISM Linkage Mapping Set version 2.5 (Applied Biosystems, Foster city, CA), within or contiguous to known adRP and adCRD genes, and within the locus *CORD12*. Results were analysed with GeneMapper software (version 4.0, Applied Biosystems, Foster city, CA). Segregation of the markers among the family members was examined.

Two-point LOD scores were calculated with Superlink-online http://bioinfo.cs.technion.ac.il/superlink-online/. The phenotype was analyzed as an autosomal dominant and fully penetrant trait with an affected allele frequency of 0.0001. Family and haplotype data were generated using Cyrillic software (version 2.1.3; Cherwell Scientific, Oxford, UK).

### SNP genotyping and analysis

To map the disease locus, a genome-wide scan was performed by the Centre National de Génotypage (CNG, http://www.cng.fr) by genotyping 262,264 SNPs (GeneChip Mapping 250 K Nsp Array, Affymetrix, Santa Clara, CA). Results were analyzed using TASE (Transmitted Allele Search Engine) a home-made software which compared every SNP between each individuals in the family.

The first test, named Common Allele to All Affected individuals (C3A), highlighted the common allele to all affected patients within the family. The second test, Transmitted Allele to All Children (TAAC), estimated the specific allele carried by the affected parent in a nuclear family (parents + child) and transmitted to the affected child. Two consecutive mismatched SNPs limited the size of the locus. Only the regions longer than 1 Mb were considered.

### Mutation screening

Coding exons and adjacent intronic sequences of candidate genes were sequenced with an Applied Biosystems 3130xL genetic analyser (Applied Biosystems, Foster city, CA) using BigDye Terminator cycle sequencing ready reaction kit V3.1 (Applied Biosystems, Foster city, CA) following manufacturer's instructions. Primer pairs and PCR conditions are available on request. Sequence analysis and mutation identification were performed using Collection and Sequence Analysis software package (Applied Biosystems, Foster city, CA).

#### Ethics Committee

Statement about Conformity with Author Information: Informed and written consent was obtained for all patients participating to the study. The study was done in adherence to the tenets of the Declaration of Helsinki.

The authors confirm that they are in compliance with their Institutional Review Boards (IRBs) as the Department of Ophthalmology of the Hospital of Montpellier has the authorization # 11018S from the French Ministry of Health for biomedical research in the field of physiology, pathophysiology, epidemiology and genetics in ophthalmology.

## Results

### Clinical description

**Figure 2:**
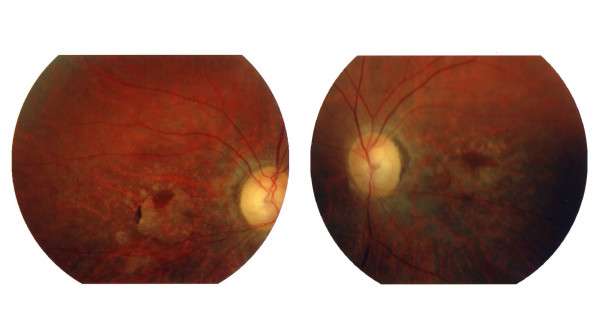
**Fundus photographs of the patient III:1**. at 47 years of age showing area of macular atrophy, rare pigment deposits and attenuation of retinal vessels.

The pedigree of the four generations family is shown in Figure 1. The 9 affected patients revealed features of adCRD with intra-familial variable phenotype including progressive loss of the visual acuity, typical bone spicule-shaped pigmentary deposits in the macular area or macular atrophy, moderate night blindness and reduced electroretinogram (ERG) responses (Table 1). The proband (III:1) showed patches of atrophy in the macular area with a few pigment deposits, attenuation of retinal arterioles and temporal pallor of the optic disc (Figure 2).

**Table 1:** Clinical features of patients with cone-rod dystrophy.

Patient	Sex	Age at onset	Symptoms	**Age at exam**.	Visual acuity OD/OS	Fundus	Visual field	ERG OD/OS Scotopic dim blue Photopic single white flash Light adapted 30-Hz flickers
**II:2**	F		None	64	20/20 20/16	Mild attenuation of retinal vessels	NA	40 μV/23 μV 181 μV/175 μV 90 μV/94 μV

**II:3**	F	40	Nystagmus Night blindness Photophobia	70	20/40 20/32	Mild attenuation of retinal vessels. Macular atrophy	OD:relative 20° central scotoma OS:absolute 20-30° central scotoma Normal PVF on both eyes	124 μV/173 μV 56 μV/60 μV 46 μV/56 μV

**III:1**	F	32	Nystagmus No photophobia No night blindness	44	20/100 20/100	Severe macular atrophy Rare bone spicule-shaped pigment deposits	Absolute 30° central scotoma and normal PVF on both eyes	48 μV/35 μV 44 μV/42 μV 32 μV/41 μV

**III:2**	M		None	38	20/25 20/20	Normal	Normal	253 μV/275 μV 30 μV/41 μV 70 μV/84 μV

**III:9**	F	35	Nystagmus Night blindness Photophobia	45	20/25 20/25	Mild attenuation of retinal vessels	Normal	130 μV/121 μV 34 μV/46 μV 41 μV/40 μV

**III:10**	M	Early childhood	Night blindness Mild photophobia	38	20/32 20/32	Posterior pole atrophy Mild attenuation of retinal vessels	Absolute 10° central scotoma and normal PVF on both eyes	157 μV/160 μV 51 μV/45 μV 88 μV/77 μV

**IV:1**	M		Photophobia	1	NA	Mild attenuation of retinal vessels. Abnormal pigmentation of the macular area.	NA	NA

**IV:4**	M		No photophobia No night blindness	11	20/32 20/50	Posterior pole atrophy Attenuated retinal vessels	Normal	NA/139 μV NA/24 μV NA/66 μV

**IV:10**	M	Early childhood	Nystagmus Photophobia No night blindness	19	20/200 20/200	Moderate pallor of the optic discs, and macular atrophy	Relative 20° central scotoma and normal PVF on both eyes	91 μV/89 μV 20 μV/13 μV 39 μV/42 μV

PVF = Peripheral Visual Field

OD/OS = oculus dexter/oculus sinister

NA = Not available

Normal value ranges are:

Scotopic dim blue: 160 μV - 250 μV

Photopic single white: 70 μV - 150 μV

Light adapted 30-Hz flickers: > 110 μV

### Mapping to CORD12

**Figure 3:**
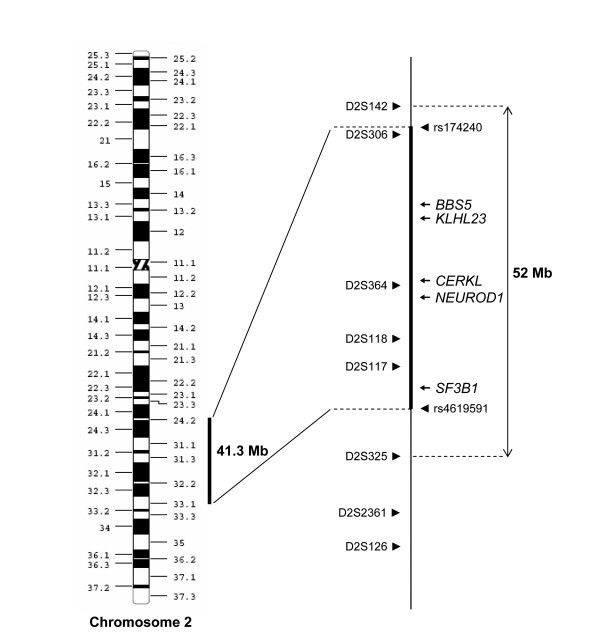
**Physical map of the linked region 2q24.2-2q33.1**. The microsatellite markers are shown on the left part of the locus. The 3 analyzed candidate genes and the 2 SNPs which delimit the locus are indicated on the right.

Microsatellite markers for the 21 adRP genes http://www.sph.uth.tmc.edu/Retnet, the 3 most frequent adCRD genes (*CRX*, *GUCY2D *and *PRPH2/RDS*) [7-10] and a fourth adCRD gene, *GUCA1A*, were used to genotype family members, and to search for co-segregation of the markers with the disease phenotype. All these candidate genes were excluded. We then performed a genome wide scan using Affymetrix 250 K microarrays and genotypes were analysed with the TASE software. No linkage was found for most chromosomal regions except for a large region located on chromosome 2q24.2-2q33.1. The boundaries of the locus were determined by SNP exclusion between SNPs rs174240 and rs4619591 and encompassed a 41.3-Mb region (Figure 3).

Microsatellite markers were then used to confirm linkage with the locus. We genotyped all 22 members of the family with 8 microsatellite markers located on 2q24.2-2q33.1 (Figure 1). All affected patients had a common haplotype and the boundaries of the region were determined by recombination events that occurred in affected individuals III:1, III:4, III:9, IV:4, IV:9 and healthy individual III:7. The proximal boundary was defined by the recombination event between markers D2S142 and D2S306 in affected patient IV:9 and distal boundary by the recombination event between markers D2S117 and D2S325 in healthy individual III:7 (Figure 3). Using Superlink software, we found a maximum LOD score of 2.86 at θ = 0 for the marker D2S118, defining a new locus named *CORD12*. The markers D2S142, D2S325, D2S2361 and D2S126, outside the locus, gave negative LOD scores (Table 2).

**Table 2:** Two-point LOD score for microsatellite markers of family RP470 calculated at different recombination fractions θ.

		Recombination fraction θ						
		
Marker	Position (Mb)	0.00	0.01	0.05	0.10	0.20	0.30	0.40
D2 S142	156,283,230	- ∞	- 0.9396	- 0.3168	- 0.1121	- 0.0002	0.0069	- 0.0000

D2 S306	160,562,440	2.6182	2.5720	2.3834	2.1389	1.6185	1.0548	0.4722

D2 S364	183,034,534	2.3172	2.2753	2.1047	1.8840	1.4168	0.9190	0.4192

D2 S118	191,606,469	2.8588	2.8094	2.6077	2.3459	1.7872	1.1789	0.5432

D2 S117	195,618,799	2.0280	1.9949	1.8600	1.6860	1.3181	0.9208	0.4865

D2 S325	208,270,870	- ∞	1.1956	1.6867	1.7184	1.4494	1.0188	0.5190

D2 S2361	216,478,443	- 3.8589	- 0.5393	0.1857	0.4554	0.5836	0.4992	0.2977

D2 S126	222,016,968	- ∞	0.2379	0.7858	0.8926	0.7898	0.5512	0.2719

The *CORD12 *41.3-Mb interval contains 280 genes. None of them were previously reported in adCRD or adRP. However the interval does contain two previously described autosomal recessive RP genes, namely *CERKL *and *BBS5*, which cause autosomal recessive RP and Bardet-Biedl syndrome, respectively [14,15]. All exons and flanking intron regions were sequenced but no mutation was found. Within the *CORD12 *locus, three other candidate genes were also sequenced. *KLHL23 *has strong similarities with the recently described gene *KLHL7 *responsible for adRP [16]. *NEUROD1 *regulates development and maintenance in the visual system [17]. *SF3B1 *is a splicing factor [18]. Other essential components of the spliceosome, *PRPF31, PRPF3, PRPF8, PAP1 *and *SNRNP200*, have been associated with adRP [19-22]. No disease causing mutations were detected in *KLHL7, NEUROD1 *and *SF3B1*.

## Discussion

In this study, a novel locus, *CORD12*, for autosomal dominant cone-rod dystrophy (adCRD) was identified and localized to chromosome 2q24.2-2q33.1. With *CORD8 *assigned to chromosome 1q23.1-q23.3, it is the second CRD locus for which the causative gene remains unknown [23]. To date, the total number of known adCRD genes and loci, including *CORD12*, is eleven.

A maximum two-point LOD score of 2.86 at θ = 0 for the marker D2S118, close to theoretical significance, was obtained. The common haplotype for affected patients in the family was flanked by SNPs between rs174240 and rs4619591, which defined the 41.3-Mb *CORD12 *locus. Two other retinal dystrophy loci are mapped on chromosome 2. *RP54*, a 19.98-Mb autosomal recessive RP interval flanked by D2S149 and D2S367 on chromosome 2p22.3-p24.1[24] and *RP28*, a 14-Mb autosomal recessive RP interval flanked by D2S1337 and D2S286 on chromosome 2p11-p15 [25,26]. The causative genes have recently been reported for both regions in September 2010, respectively *ZNF513*[27] for *RP54 *and *FAM161A *for *RP28 *[28,29]. A third gene, *C2ORF71*, was identified earlier this year next to *ZNF513*, by homozygosity mapping in two independent studies in an 8-Mb locus on chromosome 2p24.1-p23.1 and in a 6.8-Mb locus on chromosome 2p23.1-p24.1 [30,31]. None of these 3 regions overlap with *CORD12*.

The *CORD12 *41.3-Mb interval contains 280 annotated genes. We sequenced five possible candidate genes. *CERKL *and *BBS5 *which cause autosomal recessive RP and Bardet-Biedl syndrome, respectively,[14,15]*KLHL23*, which has strong similarities with the recently described gene *KLHL7 *responsible for adRP,[16]*NEUROD1 *which regulates development and maintenance in the visual system[17] and the splicing factor *SF3B1*[18]. No mutation was found in the coding region and splice sites junctions, indicating that these genes do not cause *CORD12*. However, mutations in other parts of the gene cannot be excluded. Indeed, a single-base substitution in dominant retinitis pigmentosa disease-causing gene, *PRPF31*, located deep within intron 13 was recently identified [32]. No other obvious candidate genes have been identified in *CORD12 *based on tissue expression pattern and function of gene products similar to known CRD genes. The comparison with additional families with cone-rod dystrophy showing linkage to this locus will be necessary to narrow the interval and to help the identification of a novel gene.

## Conclusions

In summary, we report on the identification of a novel locus for adCRD in chromosome 2q24.2-2q33. Identification of the disease causing gene in the interval will increase our understanding of the causes of cone-rod dystrophy.

## Competing interests

The authors declare that they have no competing interests.

## Authors' contributions

GM carried out the molecular genetic studies and the sequence alignment, participated in the design of the study and drafted the manuscript. MH performed the genotyping analysis. BB participated in the molecular genetic studies. IM participated in the medical examinations. DCC participated in the molecular genetic studies. AS participated in the sequence alignment. ABA performed the genotyping analysis. DZ performed the genotyping analysis. CPH carried out the medical examinations, conceived of the study, and participated in its design and coordination and helped to draft the manuscript. All authors read and approved the final manuscript.

## Pre-publication history

The pre-publication history for this paper can be accessed here:

http://www.biomedcentral.com/1471-2350/12/54/prepub
